# Comparing machine learning algorithms for predicting COVID-19 mortality

**DOI:** 10.1186/s12911-021-01742-0

**Published:** 2022-01-04

**Authors:** Khadijeh Moulaei, Mostafa Shanbehzadeh, Zahra Mohammadi-Taghiabad, Hadi Kazemi-Arpanahi

**Affiliations:** 1 Medical Informatics Research Center, Institute for Futures Studies in Health, Kerman University of Medical Sciences, Kerman, Iran; 2 Department of Health Information Technology, School of Paramedical, Ilam University of Medical Sciences, Ilam, Iran; 3 Department of Health Information Management, School of Health Management and Information Sciences, Kerman University of Medical Sciences, Kerman, Iran; 4 Department of Health Information Technology, Abadan University of Medical Sciences, Abadan, Iran; 5 Student Research Committee, Abadan University of Medical Sciences, Abadan, Iran

**Keywords:** COVID-19, Coronavirus, Machine learning, Artificial intelligence, Prediction hospital mortality

## Abstract

**Background:**

The coronavirus disease (COVID-19) hospitalized patients are always at risk of death. Machine learning (ML) algorithms can be used as a potential solution for predicting mortality in COVID-19 hospitalized patients. So, our study aimed to compare several ML algorithms to predict the COVID-19 mortality using the patient’s data at the first time of admission and choose the best performing algorithm as a predictive tool for decision-making.

**Methods:**

In this study, after feature selection, based on the confirmed predictors, information about 1500 eligible patients (1386 survivors and 144 deaths) obtained from the registry of Ayatollah Taleghani Hospital, Abadan city, Iran, was extracted. Afterwards, several ML algorithms were trained to predict COVID-19 mortality. Finally, to assess the models’ performance, the metrics derived from the confusion matrix were calculated.

**Results:**

The study participants were 1500 patients; the number of men was found to be higher than that of women (836 vs. 664) and the median age was 57.25 years old (interquartile 18–100). After performing the feature selection, out of 38 features, dyspnea, ICU admission, and oxygen therapy were found as the top three predictors. Smoking, alanine aminotransferase, and platelet count were found to be the three lowest predictors of COVID-19 mortality. Experimental results demonstrated that random forest (RF) had better performance than other ML algorithms with accuracy, sensitivity, precision, specificity, and receiver operating characteristic (ROC) of 95.03%, 90.70%, 94.23%, 95.10%, and 99.02%, respectively.

**Conclusion:**

It was found that ML enables a reasonable level of accuracy in predicting the COVID-19 mortality. Therefore, ML-based predictive models, particularly the RF algorithm, potentially facilitate identifying the patients who are at high risk of mortality and inform proper interventions by the clinicians.

## Background

In December 2019, the novel coronavirus disease (COVID-19) was detected in Wuhan District [1], Republic of China (ROC). Ever since, this virus has rapidly spread all over the world. In January 2020, World Health Organization (WHO) declared this outbreak as a pandemic [2, 3]. The clinical outcomes of the virus ranged from asymptomatic or mild symptoms to serious complications and, consequently, death in some cases. COVID-19 is a highly contagious viral infection and, thus far, continues to spread aggressively worldwide and has become a serious global health concern. Rapid spread of COVID-19 has resulted in the severe shortage of medical resources and exhaustion of frontline healthcare workers [4–9]. Moreover, many COVID-19 patients exacerbate rapidly after a period of quite mild symptoms, stressing the call for advanced risk stratification models. Applying predictive models can identify patients who are at the increased risk of mortality and provide support to reduce deaths as soon as possible [10–15]. Hence, to mitigate the burden on the healthcare system and provide the best care for patients, it is necessary to predict the prognosis of the disease and effectively triage critically the ill patients. Besides, due to the great hesitation surrounding its concluding influence, clinicians and health policymakers have commonly used and depended upon predictions made by different computational and statistical models [16, 17].

In response to the above-mentioned challenges, healthcare systems across the world attempt to leverage machine learning (ML) classifiers for achieving proper decision-making via eliminating physicians’ subjective evaluations [11, 18, 19]. ML as a branch of artificial intelligence (AI) enables extracting high-quality predictive models from the mining of huge raw datasets [20]. It is a valuable tool that is even more employed in medical research to improve predictive modeling and reveal new contributing factors of a specific target outcome [20, 21]. ML algorithms can reduce uncertainty and ambiguity by offering evidence-based medicine for risk analysis, screening, prediction, and care plans; they support reliable clinical decision-making and hope to improve patient outcomes and quality of care [22, 23].

This study aimed to develop a mortality risk prediction model for COVID-19 based on ML algorithms that utilize patients’ routine clinical data. We are mostly looking for the following questions: (1) What are the most relevant predictors of mortality among COVID-19 in-hospital patients? (2) What is the best ML algorithm for developing the mortality prediction model?

## Methods

### Feature identification and patient selection

This stage contained the identification of the proposed features in predicting mortalities in the patients with COVID-19. At the first step, the most relevant clinical features were determined using an extensive literature review in scientific databases. Then, a questionnaire was designed through derived features (predictors) that belonged to the patient’s demographics, risk factors, clinical manifestation, laboratory tests, and therapeutic classes. The content validity of the questionnaire was assessed by an expert panel including two infectious diseases specialists and two virologists. In addition, a test–retest (at 10-day interval) was done to evaluate the reliability of the questionnaire. Finally, the proposed clinical features were validated using a two-round Delphi survey by a group of multidisciplinary expert team, including five infectious diseases specialists, three epidemiologists, and two virologists. The experts were asked to review the initial list of the parameters to score each item according to their importance in predicting COVID-19 mortality based on a 5-point Likert scale, ranging from 1 to 5, where 1 indicates “not important” and 5 indicates “highly important”. Only the features with the average score of 3.75 (70%) and higher were allowed in the study.

**Fig.1:**
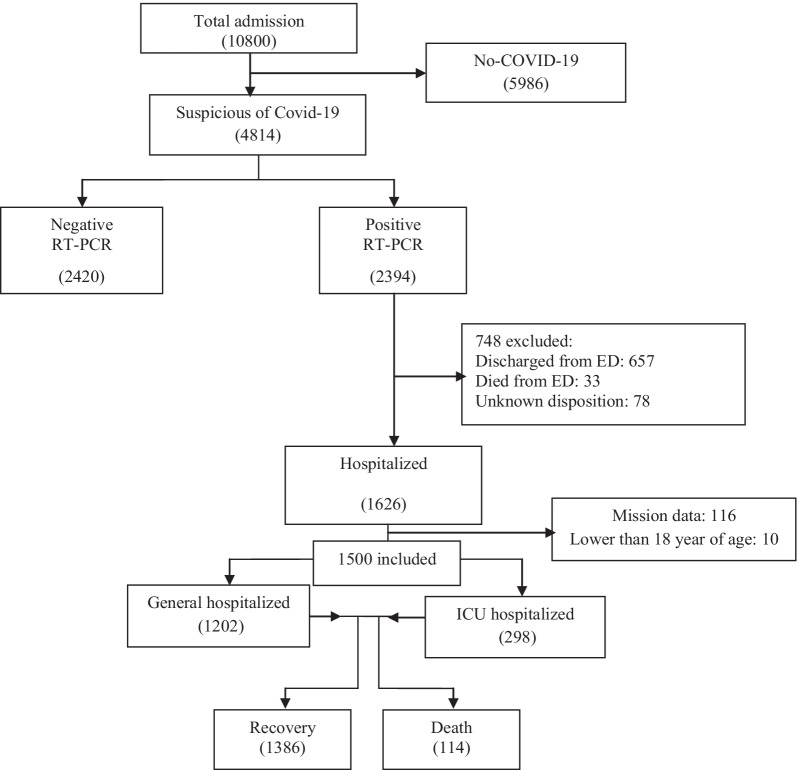
Flowchart describing patient selection

After performing a literature review coupled with a two-round Delphi survey, based on finalized feature set, data from laboratory-confirmed COVID-19 hospitalized patients (n = 1500) were extracted from a database registry in Ayatollah Taleghani Hospital, affiliated to Abadan University of Medical Sciences, which is a central hospital for COVID-19 diagnosis and treatment in the southwest of Khuzestan Province, Iran. The time frame of this study was from February 9, 2020, to December 20, 2020. During this period, 10,800 suspected cases with COVID-19 were referred to Ayatollah Taleghani Hospital’s Ambulatory and Emergency Departments. Of those, 2,394 cases were introduced as confirmed COVID-19 by real-time polymerase chain reaction (RT-PCR) test. Only the hospitalized patients who were diagnosed with positive RT-PCR tests were included in our study (see Fig. 1). All the data were checked by two health information management experts (MSH and HK-A) and a third researcher (KHM) adjudicated any variance in interpretation between the two primary reviewers. For different interpretations and missing data, we contacted the physicians who completed the form and the patient or their family members to review and supplement data. Finally, all the collected data were entered into an Excel file.

### Outcome variable

The outcome variable was deceased representing in-hospital mortality with COVID-19 and had a twofold distribution: “Yes” if the patient is deceased or “No” otherwise.

### Preprocessing

Patients who were lower than 18 years of age were excluded. These patients should be included in the scope of pediatric exploration. Patients discharged from the emergency department were excluded because their outcomes were unknown. We obtained de-identified data from 1626 patients in the Abadan CoV registry database. 116 incomplete case records which had many missing data (more than 70%) were excluded from the investigation. Also, the remaining missing values were imputed with the mean or mode of each variable. Noisy and abnormal values, errors, duplicates, and meaningless data were checked by two health information management (HIM) experts (MSH and HKH) in collaboration with two infectious diseases and hematology specialists. For different interpretations about data preprocessing, we contacted the corresponding physicians. The final sample size used in this analysis was 1,500 hospitalized patients who were over 18 years old. For detailed exclusions, a schematic of all the study inclusion criteria is shown in Fig. 1.

### Data balancing

One of the key barriers to ML algorithms is the imbalanced data problem. This occurs when the classes are not categorized equally. In the selected dataset, the amount of data in outcome classes is significantly imbalanced and it contains more samples related to the alive class (1386 cases), while the death class is much smaller (only 114 cases). Accordingly, the trained models are often delivering prejudiced results towards overriding class and the ML models are much more possible to categorize new observations to the majority class. In this study, to handle the class imbalance, the synthetic minority over-sampling technique (SMOTE) method was used in the imbalanced-learn toolbox to balance the dataset (https://imbalanced-learn.org/stable/).

### Feature selection

Feature selection is a technique commonly used in forecasting, pattern recognition, and classification modeling to lessen the dimensions and intricacy of the dataset by discarding irrelevant and redundant features. In this study, feature selection was performed to set up a model and order the input features according to their importance concerning the specific problem or target classes [20, 24]. Many feature selection methods have been suggested to select suitable features for predictive models, including Information GainRatio Attribute evaluation (GA), Forward Elimination, Backward Elimination, and One Rule Attribute Evaluation (ORAE) [25]. In this study, the GA method in Waikato Environment for Knowledge Analysis (Weka) (v3.9.2) software was used to select the features. This method measures the importance of features with respect to target class on the basis of gain ratio [25, 26]. It can be calculated by the following formula,

$${\text{GainR}}\,( {{\text{Class}}, \,{\text{feature}}} ) = ( {{\text{H}}( {{\text{Class}}} )-{\text{H }}( {{\text{Class}}|{\text{feature}}} )} )/{\text{H}}( {{\text{feature}}} )$$

where Class = Binary outcome (DN/absence of nephropathy), features = Evaluated parameter (BP, GFR, HbA1c, UACR, etc.), also seen as risk factor.

### Model development

The predictive classifier models were developed for accurately predicting COVID-19 mortality. In the modeling stage, to select the appropriate ML algorithms, the related studies in this field were reviewed [10, 11, 15, 18, 23, 27–31] along with considering the type and quality of the selected dataset. To construct the mortality prediction model, we applied seven ML algorithms including the J48 decision tree, random forest (RF), k-nearest neighborhood (k-NN), multi-layer perceptron (MLP), Naïve Bayes (NB), eXtreme gradient boosting (XGBoost), and logistic regression (LR). The SPSS software (version 23) was used to analyze the data. Finally, the algorithms were implemented using Weka (v3.9.2) software to analyze and calculate the curves and criteria, and draw the confusion.

### Cross-validation

We apply WEKA’s EXPLORER module to determine the optimal hyperparameters for all models used. The hyperparameters selected were those that attained the best performance values. A tenfold cross-validation process system was used for evaluating the performance and general error of whole classification models. By using WEKA’s EXPERIMENTER module, running all models ten times, and using repeated tenfold cross-validation, to ease comparing of the predictive performance based on the various evaluation measures that are available in WEKA [32, 33]. In tenfold cross-validation process, the original samples are randomly partitioned into 10 sub-samples of about equal size. One of the 10 sub-samples was applied as the validation dataset for testing the models, and the remaining 9 sub-samples were applied as training datasets. The cross-validation method was then repeated 10 times with one of the 10 sub-samples applied sequentially for each validation. Finally, the validation results from ten experimental models are then mixed to render the performance metrics (sensitivity, specificity, accuracy, precision, and ROC) derived from testing only [34, 35]. In the other hand, the results of the performance metrics are computed as an average of these ten runs [36–39]. In general, stratified tenfold cross-validation is proposed for estimation accuracy as a result of its relatively low-level bias and variety.

It should be noted that the ten-fold cross-validation is a widely applied and preferred validation technique in machine learning and data mining due to differing from the conventional split instance method. This method assisted to reduce the deviation in prediction error; increases the use of data for both training and validation, without overfitting or overlap between the test and validation data; and protectors against experiment theory proposed by arbitrarily split data [40].

### Model evaluation

Model performance evaluation is a fundamental part of building an effective ML model. The predictive models were evaluated using confusion matrix performance metrics (Table 1). In order to evaluate the predictive models, we applied some evaluation measures metrics including accuracy, specificity, precision, sensitivity, and receiver operating characteristic (ROC) chart criteria to measure the model’s performance. Finally, all these evaluation criteria were compared in terms of the performance to get the best model for predicting the COVID-19 moralities (Table 2).

**Table 1:** Confusion matrix

Output	Predicted values	
	Death (+)	Survive (−)
Actual value		
Death (+)	TP	FN
Survive (−)	FP	TN

True Positive = the number of cases that are truly classified as positive by the algorithm

False Positive = the number of cases that are falsely classified as positive by the algorithm

False Negative = the number of cases that are falsely classified as negative by the algorithm

True Negative = the number of cases that are truly classified as negative by the algorithm

**Table 2:** The performance evaluation measures

Performance criteria	Calculation
Accuracy	(TP + TN) /(TP + TN + FP + FN)
Precision	TP/(TP + FP)
Sensitivity/ Recall	TP/(TP + FN)
Specificity	TN/(TN + FP)

True Positive (TP), True Negative (TN), False Positive (FP), False Negative (FN)

### Ethical considerations

This study was approved by Ethical Committee Board, Abadan University of Medical Sciences (code: IR.ABADANUMS.REC.1400.008). In order to protect the privacy and confidentiality of the patients, we concealed the unique identifying information of all the patients in the process of data collection.

## Results

The results of the six stages of the study are presented below:

### Feature identification and selection

After conducting a comprehensive literature review along with a two-round Delphi survey, 54 clinical features were identified as probable predictors for determining the mortality risk of COVID-19 patients. In the next step, the degree of each factor in predicting COVID-19 hospitalized mortality based on GA method evaluation was calculated. Based on this method of 54 clinical features that remained until this step, 16 features were excluded from the study and 38 predictors were chosen as the input for the ML algorithms (Table 3). These features were divided into six categories, including demographics, risk factors, clinical manifestations, laboratory tests, and therapeutic plans.

**Table 3:** Identifying the initial list of features affecting mortality in patients with COVID-19

Classes	Number of suggested features	Delphi round		Final features	Included features	Excluded features
		< 75%	75% <			
Demographic	9	6	3	3	Gender, age, length of hospitalization	Body mass index, blood group, marital status, ethnicity, place of birth, level of education
Risk factors	10	2	7	7	Smoking, ICU admission, hypertension, pneumonia, diabetes, cardiac disease, another underline disease	Recent travel, exposure type
Clinical manifestations	23	9	14	14	Dyspnea, sore throat, runny nose, loss of taste, loss of smell, contusion, muscular pain, chill, fever, cough, nausea/ vomiting, chest pain and pressure, headache, gastrointestinal symptoms	Weakness, sneezing, exudative pharyngitis, mucus or phlegm, conjunctivitis, hemoptysis, anorexia, dry mouth, decrease consciousness
Laboratory results	24	11	13	13	Blood urea nitrogen, white cell count, C-reactive protein, hypersensitive troponin, glucose, erythrocyte sedimentation rate, creatinine, alkaline phosphatase, aspartate aminotransferase, alanine aminotransferase, absolute lymphocyte count, absolute neutrophil count,	Hematocrit, red cell count, hemoglobin, total bilirubin, thromboplastin time, prothrombin time, albumin calcium, phosphorus, magnesium, sodium, potassium
Therapeutic plan	1	0	1	1	Oxygen therapy	

In the selected feature list, dyspnea and platelet count with the correlation coefficient of 0.5532 and 0.0210, respectively, gained the highest and lowest importance for predicting the COVID-19 mortality (Table 4).

**Table 4:** Features affecting predicting mortality in patients with COVID-19

Row	Features name	Degree of importance	Row	Features name	Degree of importance
1	Dyspnea	0.5532	21	Chest pain and pressure	0.2256
2	ICU admission	0.5409	22	Absolute neutrophil count	0.2123
3	Oxygen therapy	0.3789	23	Headache	0.1992
4	Age	0.3207	24	Gender	0.186
5	Fever	0.3142	25	Gastrointestinal symptoms	0.1802
6	Cough	0.3072	26	White cell count	0.1702
7	Loss of taste	0.2944	27	C-reactive protein	0.1574
8	Loss of smell	0.2923	28	Hypersensitive troponin	0.1428
9	Hypertension	0.2768	29	Pneumonia	0.1066
10	Contusion	0.2744	30	Glucose	0.0906
11	Muscular Pain	0.2731	31	Erythrocyte sedimentation rate	0.0826
12	Chill	0.2537	32	Creatinine	0.0716
13	Runny noise	0.2532	33	Alkaline phosphatase	0.0678
14	Blood urea nitrogen	0.2524	34	Length of hospitalization	0.0626
15	Diabetes	0.2506	35	Aspartate aminotransferase	0.0445
16	Sore throat	0.25	36	Smoking	0.0427
17	Absolute lymphocyte count	0.2339	37	Alanine aminotransferase	0.0319
18	Nausea/vomiting	0.2301	38	Platelet count	0.0210
19	Other under line disease	0.2282	39		
20	Cardiac disease	0.2274			

### Patient selection

We obtained data from 1626 patients in the Abadan CoV registry database. One hundred and sixteen incomplete records which had many missing data (more than 70%) were excluded from the analysis. The final sample size used in this analysis was 1500 adult patients (over 18 years old) who were hospitalized in the hospital. For the detailed exclusions, see Fig. 1. It should be noted that Fig. 1 was designed and created by the authors according to the steps taken to select the patients.

### Participants’ characteristics

After applying the exclusion criteria and quantitative analysis of case records, the number of 1500 hospitalized COVID-19 patients met eligibility. 836 patients (55.74%) were male and 664 (44.26%) were women, and the median age of the participants was 57.25 years old (interquartile 18–100). In total, 298 (19.87%) were hospitalized in ICU and 1202 (80.13%) were in general wards. Of these, 1386 (92.4%) recovered and 114 (7.6%) deceased (Table 5).

**Table 5:** Descriptive statistics of the Features

Features (quantitative)	Range	Mean (SD)
Age (year)	18–100	57.25 (17.8)
Leng of hospitalization	1–32	61.89 (13.25)
Creatinine (mg/dL)	0.1–17.9	51.39 (14.4)
White-cell count	1300–63,000	82.34 (4897.4)
Platelet count	108,000–691,000	66.2 (38.1)
Absolute lymphocyte count	2–95	23.74 (11.8)
Absolute neutrophil count	8–98	74.52 (12.3)
Blood urea nitrogen	0.5–251	42.52 (31.7)
Aspartate aminotransferase	3.8–924	44.45 (53.5)
Alanine aminotransferase	2–672	38.29 (41.6)
Glucose	18–994	36.09 (74.2)
Lactate dehydrogenase	4.6–6973	55.68 (339.0)
Prothrombin time	0.9–46.8	42.82 (23.9)
Alkaline phosphatase	9.6–2846	21.12 (39.2)
Erythrocyte sedimentation rate	2–258	40.65 (28.8)

Features (qualitative)	Values	Frequencies
Gender	Male, Female	836, 664
Cough	Yes, No	736, 764
Contusion	Yes, No	363, 1137
Nausea/vomiting	Yes, No	802, 848
Headache	Yes, No	899, 601
Gastrointestinal symptoms	Yes, No	976, 524
Muscular pain	Yes, No	1021, 479
Chill	Yes, No	878, 622
Fever	Yes, No	728, 772
Pneumonia	Yes, No	1044,456
Oxygen therapy	Yes, No	1053, 447
Dyspnea	Yes, No	1078, 422
Loss of taste	Yes, No	272, 1228
Loss of smell	Yes, No	1195, 305
Runny Noise	Yes, No	637, 863
Sore throat	Yes, No	444, 1056
Other underline disease	Yes, No	360, 1140
Cardiac disease	Yes, No	118, 1382
Hypertension	Yes, No	395, 1105
Diabetes	Yes, No	268, 1232
Smoking	Yes, No	141, 1359
Alcohol addiction	Yes, No	26, 1474
C-reactive protein	Positive, Negative	1163, 337
Oxygen therapy	Yes, No	363, 1137
ICU admission	Yes, No	298, 1202
Deceased	Yes, No	114, 1386

### Developing and evaluating models

**Fig. 2:**
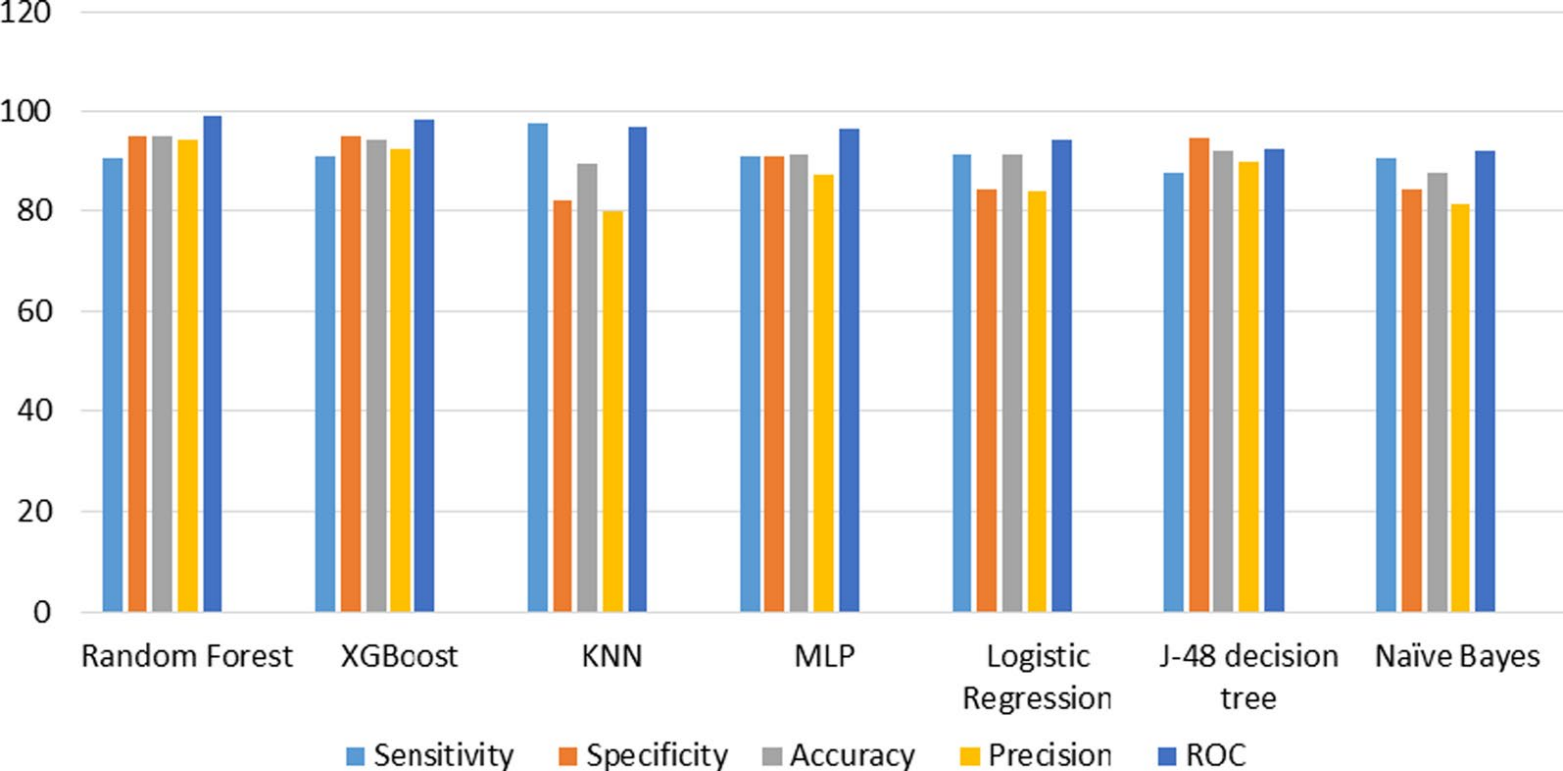
Visual comparisons of ML algorithm capabilities for COVID-19 death prediction

After selecting the best feature subset, we used various ML algorithms to build a predictive model. In this research, seven ML algorithms, including J48, MLP, XGBoost, RL, k-NN, RF, and NB, were trained for developing COVID-19 mortality prediction models. Then, the performance of each developed model was evaluated using sensitivity, specificity, accuracy, precision, and ROC of the performance metrics (Table 6). As shown in this table, the RF algorithm reaching 90.70% sensitivity, 95.10% specificity, 95.03% accuracy, 94.23% precision, and ROC value of 99.02% yielded better capability in predicting COVID-19 in-hospital mortality than other ML algorithms. Figure 2 depicted the performance metrics of the selected ML algorithms.

**Table 6:** Performance evaluation of the selected ML algorithms for COVID-19 death prediction

Algorithms	Sensitivity (%)	Specificity (%)	Accuracy (%)	Precision (%)	ROC (%)
Random forest	90.70	95.10	95.03	94.23	99.02
XGBoost	90.89	95.01	94.25	92.43	98.18
KNN	97.38	82.15	89.56	80.11	96.78
MLP	90.81	91.07	91.25	87.19	96.49
Logistic regression	91.45	84.47	91.23	83.94	94.22
J48 decision tree	87.77	94.47	92.17	89.97	92.19
Naïve Bayes	90.44	84.31	87.47	81.32	92.05

**Fig. 3:**
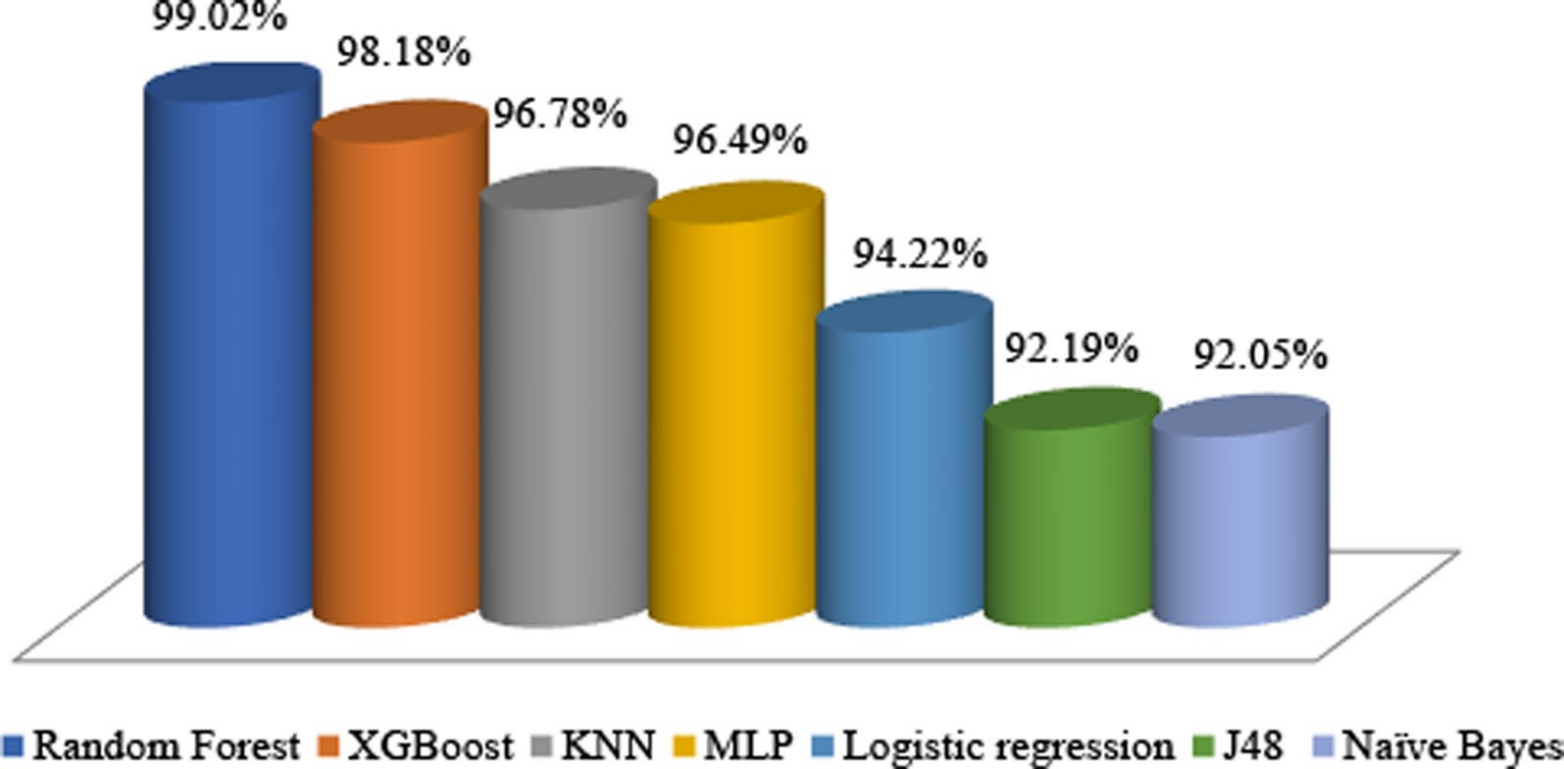
ROC chart of selected ML algorithms

The results of comparing the area under the ROC curve for the selected ML algorithms are shown in Fig. 3.

Also, based on the ROC, the Naïve Bayes algorithm attained the worst performance with the sensitivity of 90.44%, specificity of 84.31%, accuracy of 87.47%, precision of 81.32%, and ROC of 92.05%.

## Discussion

The current study aimed to retrospectively develop and validate ML models based on the most relevant features in determining the risk of COVID-19 mortality derived from extensive literature review coupled with a two-round Delphi survey. For this aim, the J48 decision tree, RF, k-NN, MLP, NB, XGBoost, and LR models were developed using a dataset of laboratory-confirmed COVID-19 hospitalized patients. The experimental results showed that RF had the best performance among the other seven ML techniques with the accuracy of 95.03%, sensitivity of 90.70%, precision of 94.23%, specificity of 95.10%, and ROC around 99.02%. Our results showed that RF, XGBoost, KNN, and MLP models have a good prediction performance, the ROC is all above 96.49%, and their diagnostic efficiency is better than the LR model trained using the same parameters.

Different studies have been evaluating the application of ML techniques in predicting mortality in the patients with COVID-19. Yadaw et al. [30] assessed the performance of four ML algorithms including LR, RF, SVM, and XGBoost using a dataset (n = 3841) for predicting COVID-19 mortality. The model developed with XGBoost happened to be the best model among all the models developed in terms of AUC with 0.91%. In another study [23] a retrospective analysis on the data of 2520 COVID-19 hospitalized patients was conducted. Results of this study showed the model developed by the neural network (NN) yielded better performance and was the best model in terms of AUC with 0.9760% in predicting COVID-19 patient's physiological deterioration and death among other models developed by logistic regression (LR), SVM, and gradient boosted decision tree. Vaid et al. [41] in their study analyzed data of 4029 confirmed COVID-19 patients from EHRs of five hospitals, and logistic regression with L1 regularization (LASSO) and MLP models was developed via local data and combined data. The federated MLP model (AUC-ROCs of 0.822%) for predicting COVID-19 related mortality and disease severity outperformed the federated LASSO regression model. Other study conducted [42] four ML techniques were trained based on 10,237 patients' data and, finally, SVM with the sensitivity of 90.7%, specificity of 91.4%, and ROC of 0.963% had the best performance. Moulaei et al. [31] also predicted the mortality of Covid-19 patients based on data mining techniques and concluded that based on ROC (1.00), precision (99.74%), accuracy (99.23%), specificity (99.84%) and sensitivity (98.25%), RF was the best model in predicting mortality. After, the RF, KNN5, MLP, and J48 were the best models, respectively [31]

In the current study, some features such as dyspnea, ICU admission, oxygen therapy (intubation), age, fever, and cough were of the highest importance; on the other hand, alcohol/addiction, platelet count, alanine aminotransferase (ALT), and smoking were of the lowest importance in predicting COVID-19 mortality. However, from the physicians' point of view, awareness of these factors may be crucial for the success of drug therapy and mortality prediction. But in ML techniques, many of these factors can be ignored from analysis and mortality can be predicted with fewer factors.

Several studies have also reported some important clinical features(predictors) for COVID-19 patient mortality by leveraging a feature analysis technique. The selected features are used as inputs for developing ML-based models for severity, deterioration, and mortality of COVID-19 patient risk analysis. The strongest predictive features included basic data such as age (aged) [11, 17, 28, 30, 43–46], gender (male) [10, 11, 18, 27, 29, 44, 46], BMI (high) [15–17], type of patient encounter (inpatient vs. outpatient) [11, 23, 27, 29], occupation (related to healthcare) [17, 23, 29, 30], clinical symptoms include dyspnea [15, 16, 23, 30, 31, 44, 47], low consciousness [11, 17, 18, 28], dry cough[15, 17, 18, 23, 27, 28, 44] fever [11, 17, 18, 43–45, 47], para-clinical indicators consisting of spo2 (decreased) [16, 18, 29, 45, 47], lymphocyte count (low) [10, 23, 27–29], platelet count (low) [16, 27–29, 47], leukocyte count (raised) [15, 16, 27, 28, 30, 44], neutrophil count (raised) [15, 23, 27, 28, 30, 43, 45], CRP (increased) [15, 29, 30, 45], D dimer (increased) [10, 30, 45], ALT and/or AST (raised) [16, 27, 28, 30, 47], cardiac troponin (increased) [23, 28, 29, 43], and LDH (elevated) [17, 27, 28, 48], and comorbidity conditions associated with poor prognosis including hypertension [28–30, 44–46], lung disease including chronic obstructive lung disease [11, 16, 27, 28], asthma [16, 18], cardiovascular disease [28–30, 43, 45, 47], cancer [11, 44, 47], pneumonia [11, 17, 46–48], and chronic renal disease [11, 15, 17, 18, 46]. On the other hand, sore throat [11, 27, 28, 30], myalgia and malaise [11, 29, 30], diarrhea and GI symptoms [23, 44, 45], and headache [11, 17, 47] for clinical manifestation and hemoglobin count [11, 15, 45, 47, 48] as well as mean cell volume (MCV) [16, 17, 28, 44] and hematocrit rate [18, 27–29] for the laboratory findings have the least importance for predicting.

Finally, ML can be of great use for the clinicians involved in treating the patients with COVID-19. The proposed algorithms can predict the mortality of the patients with optimum ROC, accuracy, precision, sensitivity, and specificity rates. This prediction can lead to the optimal use of hospital resources in treating the patients with more critical conditions and assisting in providing more qualitative care and reducing medical errors due to fatigue and long working hours in the ICU. Designing a valid predictive model may improve the quality of care and increase the survival rate of the patients. Therefore, predictive models for mortality risk analysis can greatly contribute to identifying high-risk patients and adopting the most effective assistive and treatment care plans. This could lead to decreasing ambiguity by offering quantitative, objective, and evidence-based models for risk stratification, prediction, and eventually episode of the care plan. It offers a better strategy for clinicians to lessen the complications and improve the likelihood of patient survival.

## Conclusion

In this study, we created and evaluated ML-based prediction models for in-hospital mortality using the most important clinical features(38 predictors). The RF model performed best on classification accuracy among the other four ML algorithms. The proposed model can be suitably used for predicting the mortality risk of hospitalized COVID-19 patients and maximizing the use of restricted hospital resources. This model could automatically identify high-risk patients as early as the time of admission or during hospitalization. In conclusion, the use of ML algorithms in combination with qualitative and comprehensive hospital databases such as patient registries can enable timely and accurate mortality risk classification of COVID-19 patients. In the future, the performance of our model will be enhanced if we test more classification techniques at larger, multicenter, and qualitative datasets.

### Limitations

Our work had several limitations that must be considered. First, this was a retrospective study design with the documented data that were irregular or imbalanced; thus, we balanced them by eliminating noise and inadequate records as much as possible from the dataset. To solve the imbalanced dataset problem, in which the number of records related to the dead class was significantly lower than the recovery or alive (144 vs 1386), different criteria were considered to measure the performance of each ML algorithm. Also, by using the SMOTE, the bias was minimized via class balancing. Another limitation was that it was conducted in a single-center registry database, which may limit the generalizability of the developed models. However, the ABADANUMS CoV registry is a database collected at a designated hospital in Abadan city that delivers special healthcare services to COVID-19 patients. Nonetheless, we will use multi-center data to perform the external validation of the proposed model for enhancing the widespread prediction. Other features concerning the lung CT or radiology images could have been included. However, consistent with the purpose of the current research, considering only the routine clinical features of the patients while being admitted would suffice. Although the constraint of using data at admission inspires the usage of the model in patient triage, events that happened during a patient hospitalization period may drive their clinical course ahead of the previous likelihood, which cannot be apprehended by routine admission features. We believed a real-time or incessantly updating modeling method would be better matched for this as a future direction. Furthermore, we do not have information about the time span from symptom beginning to admission, which might have had an influence on the features that we sampled on hospital admission. Thus, the dynamic variations of some significant features must be followed up to better and timely recognize patients at higher risks of poor outcomes.

Finally, in the present study, patients who were less than 18 years of age and patients discharged from the emergency department were excluded from the study. If these people were included in the study, different results might have been obtained.

## Data Availability

All data generated and analyzed during the current study are not publicly available but are available from the corresponding author on reasonable request and the Abadan University of Medical Sciences' approval.

## Abbreviations

ML: Machine learning
RF: Random forest
k-NN: K-nearest neighborhood
MLP: Multi-layer Perceptron
NB: Naïve Bayes
ROC: Receiver operating characteristic
WHO: World Health Organization
AI: Artificial Intelligence
RT-PCR: Real-time polymerase chain reaction
TP: True Positive
TN: True Negative
FP: False Positive
FN: False Negative
WEKA: Waikato Environment for Knowledge Analysis
XGBoost: EXtreme gradient boosting
NN: Neural network
LR: Logistic regression
LASSO: Logistic regression with L1 regularization
MCV: Mean cell volume
SMOTE: Synthetic minority over-sampling technique
